# Defining the interconnectivity of the medial prefrontal cortex and ventral midbrain

**DOI:** 10.3389/fnmol.2022.971349

**Published:** 2022-07-22

**Authors:** May Hui, Kevin T. Beier

**Affiliations:** ^1^Department of Physiology and Biophysics, University of California, Irvine, Irvine, CA, United States; ^2^Department of Pharmaceutical Sciences, University of California, Irvine, Irvine, CA, United States; ^3^Department of Biomedical Engineering, University of California, Irvine, Irvine, CA, United States; ^4^Department of Neurobiology and Behavior, University of California, Irvine, Irvine, CA, United States; ^5^Center for the Neurobiology of Learning and Memory, University of California, Irvine, Irvine, CA, United States; ^6^UCI Mind, University of California, Irvine, Irvine, CA, United States

**Keywords:** prefrontal cortex, dopamine, ventral midbrain, Rabies, connectivity

## Abstract

Dysfunction in dopamine (DA) signaling contributes to neurological disorders ranging from drug addiction and schizophrenia to depression and Parkinson’s Disease. How might impairment of one neurotransmitter come to effect these seemingly disparate diseases? One potential explanation is that unique populations of DA-releasing cells project to separate brain regions that contribute to different sets of behaviors. Though dopaminergic cells themselves are spatially restricted to the midbrain and constitute a relatively small proportion of all neurons, their projections influence many brain regions. DA is particularly critical for the activity and function of medial prefrontal cortical (mPFC) ensembles. The midbrain and mPFC exhibit reciprocal connectivity – the former innervates the mPFC, and in turn, the mPFC projects back to the midbrain. Viral mapping studies have helped elucidate the connectivity within and between these regions, which likely have broad implications for DA-dependent behaviors. In this review, we discuss advancements in our understanding of the connectivity between the mPFC and midbrain DA system, focusing primarily on rodent models.

## Introduction

The prefrontal cortex (PFC) is critical for a variety of executive functions, including motivation, attention, decision-making, inhibitory control, and memory (Toshiyuki and Goldman-Rakic, 1991; Miller, 1999; Bechara et al., 2000; Ridderinkhof et al., 2004; Euston et al., 2012; Laubach et al., 2015). In rodents, deficits in PFC signaling have been linked to impaired performance on memory acquisition and consolidation (Bontempi et al., 1999; Tronel and Sara, 2003). Likewise, deficits in homologous regions in primates, such as the dorsolateral PFC and anterior cingulate cortices, interfere with memory and decision making (Amiez et al., 2006; Oyama et al., 2021).

Proper functioning of the medial prefrontal cortical (mPFC) hinges upon a delicate balance of neuromodulator input. Among these, the most well-characterized neuromodulators include acetylcholine (ACh), serotonin (5-HT), norepinephrine (NE), and dopamine (DA), each of which exerts important and varied effects on mPFC function. For example, disruptions to cholinergic input from the basal forebrain have detrimental outcomes on attention (Robbins et al., 1989; Pang et al., 1993; Voytko et al., 1994; McGaughy et al., 2002; Dalley, 2004; Newman and McGaughy, 2008; Hasselmo and Sarter, 2011), whereas impaired serotonergic innervation from the medial and dorsal raphe nuclei has opposing effects (Schmitt et al., 2000; Gallagher et al., 2003; Wingen et al., 2007). In contrast, NE and DA appear to have synergistic effects – prior studies have suggested that prefrontal cortical NE transmission is necessary for DA release in the mPFC (Ventura et al., 2003, 2005, 2007), and DA can be taken up through the NE transporter (Sesack et al., 1998; Morón et al., 2002). Disruptions in the transmission of either neuromodulator lead to impairments in working memory (Brozoski et al., 1979; Williams and Goldman-Rakic, 1995; Arnsten, 2004; Ramos and Arnsten, 2007; Vijayraghavan et al., 2007; Levy, 2009; Arnsten and Jin, 2014). Altogether, the differential input of these neuromodulators likely helps to fine-tune prefrontal processing in a context-dependent manner.

Each of these four neuromodulators innervate overlapping but distinct spatial domains of the mPFC, with significant anatomical differences across species (Berger et al., 1991; Coppola et al., 2018). Both factors likely impact subsequent behavioral differences in the effects of different neuromodulators. For example, locus coeruleus (LC) NE neurons in the brainstem broadly target the entire cerebral cortex and serve as the main source of NE transmission to the mPFC. DA innervation, on the other hand, is much more spatially restricted, with midbrain ventral tegmental area (VTA) cells primarily innervating the mPFC. Expansion of this dopaminergic midbrain-to-cortical, or “mesocortical pathway,” in higher-order mammals is thought to play a role in the increasing complexity of decision-making skills found in higher-order mammals. Primates exhibit larger, re-organized terminal fields, different co-localization of neuropeptides, and developmental differences in circuit maturation relative to rodents (Berger et al., 1991). In this mini-review, we will focus primarily on viral mapping studies linking the mesocortical DA system and its downstream target, the mPFC.

## Role of dopamine in the medial prefrontal cortex

Of the major dopaminergic pathways in the brain, the mesocorticolimbic system, which comprises the mesocortical and mesolimbic system, is a key regulator of motivation, reward, and aversive behavioral responses. The mesolimbic pathway, which transmits DA from the midbrain to the ventral striatum, has historically garnered more attention from the scientific community; its role in reward-related cognition is well-characterized. In contrast, the mesocortical limb is less well-understood, but has been implicated in behavioral responses to aversive cues (Thierry et al., 1976; Abercrombie et al., 1989; Mantz et al., 1989) and therefore serves as a promising target for understanding how integration of different environmental cues can influence behavioral outcomes in the face of aversive stimuli.

Dopamine signaling within the mPFC has been shown to play a role in a variety of neuropsychiatric disorders, such as schizophrenia, post-traumatic stress disorder, and attention deficit hyperactivity disorder (Deutch and Young, 1995; Okubo et al., 1997; Lindström et al., 1999; Granon et al., 2000; Arnsten and Dudley, 2005; Howes and Kapur, 2009; Pitman et al., 2012; Grace, 2016; Lee et al., 2016). However, defining the exact signals carried by DA in the mPFC has proven to be complicated, as studies have shown that mesocortical DA cells are activated by both aversive and rewarding stimuli (Thierry et al., 1976; Mantz et al., 1989; Finlay et al., 1995; Ahn and Phillips, 1999; Bassareo et al., 2002; Lammel et al., 2011; St. Onge et al., 2012; Kim et al., 2016; Ellwood et al., 2017). Moreover, while the overall anatomy of the dopaminergic system is well-preserved across all mammals and birds, there are differences between species that may give rise to species-specific functions. Given the heterogeneity of cell types, neurotransmitters, receptor expression, and projections within both the mPFC and ventral midbrain, careful mapping of each structure may provide insight into how communication with one another regulates behavior.

## Features of VTA^DA^→mPFC cells

Midbrain DA cells project to a variety of forebrain sites, including the nucleus accumbens (NAc), dorsal striatum, amygdala, and mPFC. In each output region, DA appears to facilitate distinct behavioral adaptations. One potential mechanism for the heterogeneity in output responses is that each output region comprises a unique combination of the five possible DA receptors. Another possibility is that separate populations of VTA^DA^ cells project to each of these forebrain sites. Studies examining DA release indicate that DA release in each of these regions is different, favoring the latter hypothesis. For example, while DA release in the NAc primarily occurs in response to rewarding stimuli, DA release in the mPFC is largely a response to aversive stimuli or stressful events (Abercrombie et al., 1989; Bassareo et al., 2002; Lammel et al., 2014). Using retrobead injections into multiple forebrain sites, retrograde mapping studies labeled distinct groups of cells in the VTA that could be defined by their projection sites, further reinforcing this hypothesis (Lammel et al., 2008). In addition to being identified by their output sites, these VTA cells have unique electrophysiological properties. DA cells projecting to the mPFC, for example, are (1) located in the medial aspect of the VTA, (2) have lower expression levels of the dopamine transporter, DAT, than DA cells projecting to the nucleus accumbens lateral shell (NAcLat) or dorsal striatum, (3) have broad action potential waveforms with no after-hyperpolarizations and a relatively high maximal firing frequency (∼25 Hz), and (4) do not express D2 autoreceptors (Lammel et al., 2008). Relative to DA cells projecting to the NAcLat, mPFC-projecting VTA^DA^ cells receive preferential inputs from the lateral habenula (LHb); moreover, activation of LHb inputs to the VTA resulted in conditioned place aversion that could be reversed by infusion of the D1 antagonist SCH23390 (Lammel et al., 2012). In addition, VTA^DA^→mPFC cells were not significantly activated in mice that received a reward but were activated following an aversive tail shock, lending further support toward the hypothesis that VTA^DA^→mPFC cells signal aversion (Kim et al., 2016).

Recently, we performed comprehensive input-output viral-genetic mapping experiments of VTA^DA^ subpopulations, including the mPFC. In contrast to the previous retrograde mapping strategy, we retrogradely targeted VTA^DA^→mPFC cells using an intersectional viral-genetic strategy and labeled neurons, including their entire axonal arbors, with GFP. Consistent with previous studies, we found that VTA^DA^→mPFC cells largely had distinct arborization patterns relative to other VTA^DA^ subpopulations. However, we did observe significant overlap, predominantly with VTA^DA^ cells projecting to the medial shell of the nucleus accumbens (NAcMed) and amygdala (Beier et al., 2019). Altogether, our results indicated that VTA^DA^ cells projecting to the mPFC, NAcMed, and amygdala share a closer set of outputs with one another than with VTA^DA^→NAcLat cells. Interestingly, this also applies to the gene expression and electrophysiological properties of these cells, as identified previously (Lammel et al., 2008).

When assessing the global input patterns of VTA^DA^→mPFC cells, we found that they were highly similar to that of VTA^DA^→NAcMed and VTA^DA^→amygdala cells, further reinforcing the similarity of these cells (Beier et al., 2015). A deeper dive into the input-output connectivity of each VTA^DA^ subpopulation suggested that VTA^DA^→mPFC cells receive preferential inputs from the dorsal raphe (DR) relative to other VTA^DA^ subtypes (Derdeyn et al., 2022). However, a logistic regression model found that the identity of VTA^DA^→mPFC cells could not be predicted well by inputs, indicating that these cells did not have a highly unique global input pattern relative to the other VTA^DA^ subpopulations. Instead, the identity of VTA^DA^→mPFC cells could be decoded relatively well by their location in the dorsomedial aspect of the VTA (Derdeyn et al., 2022). This means that while VTA^DA^→mPFC cells share a high degree of similarity with VTA^DA^→NAcMed and VTA^DA^→amygdala cells, they possess several distinctive features, such as their preferential response to aversive stimuli and lack of D2 autoreceptors.

## Species differences between rodents and primates in VTA^DA^→mPFC cells

**FIGURE 1:**
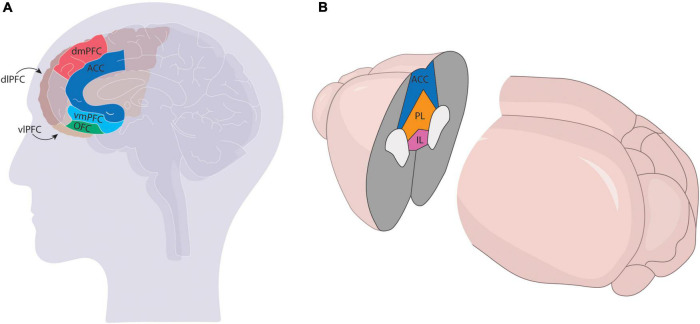
Comparison of mPFC in the primate **(A)** and rodent **(B)** brains. The mPFC has more subdivisions in primates [ventrolateral (vlPFC), ventromedial (vmPFC) dorsolateral (dlPFC), dorsomedial (dmPFC), anterior cingulate (ACC), and orbitofrontal (OFC)] and comprises a larger region of the cortex than the rodent brain, which generally only comprises the infralimbic (IL), prelimbic (PL), and ACC regions.

Relative to the VTA, the PFC is dramatically expanded in primates compared to rodents. Most of this increase stems from the expansion of sensory association cortices, which is thought to have contributed to higher-level information processing and therefore more complex behavioral outcomes (Pandya and Yeterian, 1996). Most neuroanatomists agree that the human PFC can be divided into the dorsolateral (dlPFC), ventrolateral (vlPFC), dorsomedial (dmPFC), ventromedial (vmPFC), anterior cingulate cortex (ACC), and orbitofrontal (OFC) regions, with some debate over the topographic mapping of subdivisions onto various Brodmann areas. Though the precise mapping of specific prefrontal nuclei found in rodent models onto the primate brain is still unclear, most agree the mPFC in rodents comprises the ACC, prelimbic cortex (PL), and infralimbic cortex (IL), though some studies also include the secondary motor cortex (M2) (Figure 1).

Though dopaminergic innervation of the cortex shares some similar features across mammalian species, the laminar distribution and extent of innervation varies significantly, in a manner that is potentially suggestive of higher-level cognitive capacity. For example, studies in cats, monkeys, and humans report a similar bilaminar pattern of D1-specific binding of [^3^H]SCH23390 in the cerebral cortex that is absent in rats (Lidow et al., 1991), though overall dopaminergic innervation of the cortex appears more homogenous across cortical layers in primates relative to rodents (Williams and Goldman-Rakic, 1993; vander Weele et al., 2018). Laminar distribution of receptors also varies across species, with cortical layer 1 expressing high dopaminergic receptor density in primates but not in rodents. In addition, primates exhibit denser dopaminergic innervation relative to rodents throughout the brain (Thierry et al., 1973; Descarries et al., 1987; Lewis and Sesack, 1997; Bentivoglio and Morelli, 2005). Regions such as the motor, premotor, and supplementary motor area are highly innervated in primates whereas the equivalent regions in rats receive little to no dopaminergic projections (Berger et al., 1991).

## Reciprocal connectivity from the medial prefrontal cortex to the midbrain

Circuit mapping studies have long pointed to reciprocal connectivity between the VTA and mPFC (Lindvall et al., 1984; Sesack and Pickel, 1992; Carr and Sesack, 1998). However, it remains unclear whether mPFC cells that receive input from VTA^DA^ cells are modulated by the same mesocortical dopamine neurons to which they project. Previous electron microscopy work examining inputs from the mPFC in the VTA of rat brains suggested that the mPFC sent inputs onto VTA^DA^→mPFC cells and VTA^GABA^→NAc cells, but not VTA^DA^→NAc cells (Carr and Sesack, 2000). However, our viral-genetic mapping experiments have found the opposite to be true – the mPFC preferentially provides input onto VTA^DA^→NAcLat cells. We observed this bias toward VTA^DA^→NAcLat cells to be true for anterior cortex inputs generally, as well as the mPFC specifically (Beier et al., 2015, 2019). This could be because VTA^DA^→NAcLat and VTA^DA^→mPFC cells are located in different areas of the VTA that were incompletely sampled in the Carr and Sesack study (Lammel et al., 2008; Beier et al., 2015), whereas RABV mapping samples broader areas of the VTA. It is also possible that whereas electron microscopy is a powerful method, it is less well-suited for large-scale connectivity analysis than RABV-based mapping. Furthermore, stimulation of frontal cortex inputs to the midbrain was reinforcing, and this reinforcement was blocked by infusion of the D1/D2 non-selective antagonist flupentixol in NAcLat, indicating that the functional output of this circuit was to trigger DA release into the NAc and not the mPFC (Beier et al., 2015). This result was supported by a separate study demonstrating that activating inputs from the frontal cortex to the midbrain was locomotor-activating and led to DA release in the striatum, and that this effect could be blocked by the D2 receptor antagonist haloperidol (Kim et al., 2015). Moreover, burst stimulation from VTA^DA^ neurons does not alter the mean firing rate of mPFC→VTA neurons, further suggesting that these neurons may not receive direct modulation from VTA^DA^ neurons (Au-Young et al., 1999). Unlike mPFC neurons, which exhibit sustained increases during the delay period of a classical delay task, primate A10 (VTA in primates) cells increased in firing rate only during the presentation of the cue light (Ljungberg et al., 1991; Schultz et al., 1993), suggesting that local mechanisms and not DA release by VTA^DA^→mPFC cells may be responsible for modulation of mPFC neurons. While this does not rule out ultrastructural connections between the mPFC and VTA^DA^→mPFC cells that were not detected by RABV mapping, that the behavioral results align with our viral-genetic mapping approach indicates that mPFC activation functionally leads to DA release in the NAc and is reinforcing.

## Medial prefrontal cortex cell inputs

Several studies have mapped the input landscape onto different sets of mPFC cells. DeNardo et al. (2015) mapped and compared inputs to layer 5 neurons between the mPFC and barrel cortex. Both regions shared a similar distribution of local inputs, though mPFC L5 neurons received both a greater proportion of inputs from layer 1 vs. layer 3 cells as well as approximately 2.5-fold greater inputs from local GABAergic interneurons than comparable cells in the barrel cortex. This increased interneuron presence suggests that mPFC L5 cells are under stronger inhibitory control – potentially important in providing feedforward inhibition onto L5 output cells.

While their local input distribution may have been similar, the long-range input connectivity of the mPFC and barrel cortex differed substantially. L5 cells in the barrel cortex received about 79% of their total inputs from local neurons, whereas the majority of inputs to mPFC L5 cells arose from non-local brain sites such as other prefrontal areas including the agranular insula, and the dorsal thalamus subcortical regions such as the amygdala and hypothalamus. In total, roughly ∼60% of inputs to the mPFC L5 cells arise from cortical inputs, though these are distributed across the frontal cortex and in the contralateral hemisphere.

More recently, Ährlund-Richter et al. (2019) mapped inputs to four different cell types in the mPFC: three interneuron types (parvalbumin, somatostatin, VIP), as well as mostly excitatory neurons defined by the expression of the CamKII promoter. As in the DeNardo study, the majority of inputs to the mPFC arose from the cortex, with additional significant input from the thalamus and hypothalamus. As is typical of viral mapping studies examining inputs from intermingled cell types, the authors observed that inputs arose from largely overlapping regions providing quantitatively similar input. However, the Ährlund-Richter study noted a much higher proportion of inputs from other PFC sites than DeNardo’s (approximately ∼50% compared to ∼18%, respectively). This discrepancy could potentially be attributed to the former’s viral strategy, which may have resulted in off-target labeling of neurons near the injection site, while the DeNardo paper used a version of TVA that limits off-target labeling in the absence of Cre recombination. Therefore, while the two studies largely agree on the identity of regions that provide direct input to the mPFC – which are consistent with older studies using classical tracers (Hoover and Vertes, 2007) – they bring into question the precise quantitative contribution of these input regions to different cell types. However, while the Ährlund-Richter study included an analysis of laminar distribution for local GABAergic interneuron interconnectivity, they did not perform the same analysis when mapping whole-brain inputs to the mPFC. This is a significant omission, as it may explain a relatively high fraction of the variance in inputs, as observed in other comparable studies (DeNardo et al., 2015; Wall et al., 2016).

## Ventral tegmental area interconnectivity

While local microcircuit connectivity onto VTA^DA^→mPFC cells has not yet been mapped, we recently performed an analysis that allowed us to link local inputs to putative VTA^DA^→mPFC cells by examining brain regions with similar variance across brains quantified for RABV labeling (Beier, 2022). Our results indicated that VTA^DA^→mPFC cells receive preferential inputs relative to other VTA^DA^ cells from several midline structures – the interpeduncular nucleus (IPN), raphe magnus, and supramammillary nucleus – as well as other regions, such as the superior colliculus, anterior tegmental nucleus, pedunculopontine tegmental nucleus, pontine reticular nucleus, reticulotegmental nucleus, microcellular tegmental nucleus, retrorubal field, and dorsal nucleus of the lateral lemniscus. Notably, this list includes inputs from neither the VTA nor the rostrotegmental nucleus, which contain GABA neurons thought to oppose the action of reward-related DA cells. Though we also identified robust DA-DA interconnectivity in the VTA, VTA^DA^→mPFC cells lack D2 autoreceptors and thus are unlikely to be strongly influenced by DA release from other DA cells. However, at this point, little is known about how local inputs to VTA^DA^→mPFC cells may contribute to their unique function.

## Open questions

There remain many unanswered questions about the role of midbrain and mPFC connections in adaptive and pathological behaviors. For one, while some of the studies discussed in this review have attempted to define the input landscape to different mPFC subpopulations, their findings were not entirely in agreement (DeNardo et al., 2015; Ährlund-Richter et al., 2019). A more careful and comprehensive analysis of mPFC cell types that includes starter cell spatial information could help resolve these discrepancies. Furthermore, it remains unclear how input connectivity to the mPFC relates to the outputs of different mPFC subpopulations. We recently developed a method, Tracing the Relationships between Inputs and Outputs (TRIO) that could be used to map the inputs to output-defined mPFC cells (Beier et al., 2015; Lerner et al., 2015; Schwarz et al., 2015).

While further connectivity mapping would certainly help define the link between the mPFC and VTA, to understand how DA influences mPFC outputs, we also need to consider the potential impact of DA receptor heterogeneity in different mPFC cell populations. All DA receptors (D1-D5) are found in the mPFC with varying levels of expression. While the broad expression patterns of DA receptors have been mapped, their relation to subtypes of glutamatergic and GABAergic neurons remains unclear. Combining viral mapping studies with single nucleus RNA sequencing could help illuminate the constellation of DA receptor expression in the mPFC. Moreover, while the mesocortical pathway is thought to be the source of DA in the mPFC, it is possible DA may also be released from NE cells in the locus coeruleus – a phenomenon recently reported in the hippocampus (Takeuchi et al., 2016). Because mesocortical DA cells largely innervate deeper layers of the mPFC whereas NE cells innervate more superficial layers, DA release from NE cells would expand the direct influence of DA throughout the mPFC.

## Author contributions

MH and KB wrote the manuscript. Both authors contributed to the article and approved the submitted version.

## Conflict of interest

The authors declare that the research was conducted in the absence of any commercial or financial relationships that could be construed as a potential conflict of interest.

## Publisher’s note

All claims expressed in this article are solely those of the authors and do not necessarily represent those of their affiliated organizations, or those of the publisher, the editors and the reviewers. Any product that may be evaluated in this article, or claim that may be made by its manufacturer, is not guaranteed or endorsed by the publisher.
